# *TRMT10A*‐Related Neurodevelopmental Disorder Without Metabolic Findings

**DOI:** 10.1155/humu/8058409

**Published:** 2026-05-22

**Authors:** Döndü Ülker Üstebay, İrem Aksu Şahin, Sefer Üstebay, Gül Ünsel Bolat, Hilmi Bolat

**Affiliations:** ^1^ Department of Pediatric Neurology, Faculty of Medicine, Bandırma Onyedi Eylül University, Balıkesir, Türkiye, bandirma.edu.tr; ^2^ Department of Medical Genetics, Faculty of Medicine, Balıkesir University, Balıkesir, Türkiye, balikesir.edu.tr; ^3^ Department of Pediatrics, Faculty of Medicine, Bandırma Onyedi Eylül University, Bandırma, Türkiye, bandirma.edu.tr; ^4^ Department of Child and Adolescent Psychiatry, Faculty of Medicine, Balıkesir University, Balıkesir, Türkiye, balikesir.edu.tr

**Keywords:** epilepsy, exome sequencing, intellectual disability, microcephaly, neurodevelopmental disorder, short stature, *TRMT10A*

## Abstract

*TRMT10A* is a tRNA methyltransferase gene associated with a rare autosomal recessive disorder characterized by microcephaly, intellectual disability, epilepsy, short stature, and abnormalities in glucose metabolism. Although an increasing number of patients have been reported, the extent of phenotypic variability and genotype–phenotype correlations remains incompletely understood. We report a 15‐year‐old male presenting with microcephaly, intellectual disability, epilepsy, and short stature, without evidence of diabetes or other metabolic abnormalities at the time of evaluation. Neurodevelopmental delay was evident from early childhood, and electroencephalography revealed generalized epileptiform activity requiring treatment, whereas brain magnetic resonance imaging was normal. Exome sequencing identified a homozygous stop‐gained pathogenic (PVS1, PM2, and PM3) variant in *TRMT10A* (c.127C>T; p.Arg43Ter), which was confirmed by Sanger sequencing and showed segregation consistent with autosomal recessive inheritance. This case represents one of the rare reported *TRMT10A*‐related syndrome patients in whom diabetes has not yet been documented. This observation highlights the clinical importance of establishing the diagnosis prior to the onset of diabetes, enabling anticipatory monitoring and timely intervention for potential metabolic complications. These findings underscore the importance of considering *TRMT10A* in the differential diagnosis of patients with microcephaly, intellectual disability, epilepsy, and growth abnormalities, and emphasize the need for longitudinal follow‐up to monitor for the possible later development of endocrine and metabolic manifestations.

## 1. Introduction

Transfer RNA methyltransferase 10 homolog A (*TRMT10A*) gene (OMIM  ^∗^616013), located on chromosome 4q23, encodes a guanosine‐specific methyltransferase responsible for catalyzing the N1‐methylguanosine (m^1^G9) modification in the ninth position of specific cytoplasmic transfer RNAs (tRNAs) [1, 2]. In eukaryotic cells, this modification is present in a substantial proportion of cytoplasmic tRNAs and plays an important role in maintaining tRNA stability and proper translational function [3, 4].

In higher eukaryotes, *TRMT10A* is considered the principal enzyme responsible for the m^1^G9 modification in multiple cytoplasmic tRNA species, including translationally critical tRNAs such as tRNA‐iMet and tRNA‐Gln, and its activity appears to be nonredundant in human cells. [1, 5, 6]. Disruption of this enzymatic function has been implicated in human disease.

Pathogenic variants in T*RMT10A* have been associated with a rare autosomal recessive neurodevelopmental syndrome characterized by microcephaly, intellectual disability, epilepsy, impaired glucose metabolism, and short stature, sometimes accompanied by growth retardation and delayed puberty [7–15]. Increasing numbers of patients with biallelic *TRMT10A* variants have been reported in recent years, expanding the phenotypic spectrum associated with this gene.

The *TRMT10A* gene is expressed in multiple tissues but shows particularly high expression in the brain and pancreatic islets. *TRMT10A* transcripts have also been detected in the liver, kidney, spleen, lung, and adipose tissue, whereas lower expression levels have been reported in the heart and skeletal muscle. [9]. This tissue distribution is consistent with the clinical manifestations of *TRMT10A*‐related syndrome, particularly neurological abnormalities and disturbances in glucose metabolism.

Although the precise role of *TRMT*10A in mammals remains incompletely understood, the gene is thought to regulate translational processes through its tRNA methyltransferase activity. [9]. Experimental studies have shown that *TRMT10A* deficiency is associated with decreased levels of tRNA‐iMet and tRNA‐Gln, which may impair translational efficiency and affect the synthesis of proteins involved in neuronal function. [1, 5]. Moreover, *TRMT10A*‐deficient mouse models exhibit reduced body weight, impaired synaptic plasticity, and deficits in learning and memory, findings that are consistent with the short stature and neurodevelopmental abnormalities reported in affected individuals [3].

The syndrome was first described by Igoillo‐Esteve et al. in three siblings presenting with early‐onset diabetes, microcephaly, and intellectual disability. Subsequent studies have expanded the phenotypic spectrum, with additional features including dysmorphic facial characteristics, growth retardation, and delayed puberty reported in some patients. [7–15].

In this study, we present a patient with a homozygous *TRMT10A* variant and compare the clinical features with previously reported patients to better characterize the phenotypic spectrum of *TRMT10A*‐related syndrome.

## 2. Materials and Methods

### 2.1. Patients

The patient was referred to the Pediatric Neurology Department for the evaluation of learning difficulties, decreased academic performance, and episodes of staring, with a suspected underlying neurological or genetic etiology. A comprehensive clinical assessment was performed, including detailed medical history, family history, and pedigree analysis. The patient underwent a thorough pediatric and neurological examination by specialists in pediatric neurology and medical genetics. Clinical evaluation included electroencephalography (EEG), brain magnetic resonance imaging (MRI), and bone age assessment, in addition to routine laboratory investigations. All clinical findings, radiological imaging results, and genetic testing data were integrated into the overall clinical assessment.

Written informed consent for genetic testing and the use of clinical data for research purposes was obtained from the patient′s legal guardians. The study was conducted in accordance with the principles of the Declaration of Helsinki, and ethical approval was obtained from the Ethics Committee of Bandırma Onyedi Eylül University (Decision No.: 26‐20, March 2026).

### 2.2. Genetic Testing

Genomic DNA was isolated from leukocytes obtained from the patient′s peripheral blood. As an initial cytogenetic evaluation, conventional karyotype analysis was performed. For this purpose, a short‐term 72‐h cell culture was established from the patient′s specimen. Metaphase chromosomes obtained from the cultured cells were stained using the GTG‐banding technique, and karyotypic analysis was performed using the ARGENIT‐AKAS analysis software.

Following conventional cytogenetic evaluation, chromosomal microarray analysis (HT‐CMA) was conducted using the CytoScan HT‐CMA 96‐Array Plate together with the Applied Biosystems HT Target Prep Reagent 96F kit (Thermo Fisher Scientific) on the GeneTitan Multi‐Channel (MC) Instrument. This microarray platform contains approximately 1,000,000 SNP probes and 130,000 nonpolymorphic probes, providing a total of 1.1 million probes for high‐resolution genome‐wide detection of copy number variations (CNVs). Raw data were processed and analyzed using Chromosome Analysis Suite (ChAS) and Reproductive Health Analysis Suite (RHAS) software packages. Genomic coordinates were aligned to the NCBI Human Genome reference build 38 (GRCh38/hg38). Detected CNVs and other genomic alterations were interpreted using publicly available databases, including DECIPHER, OMIM, ClinVar, and other relevant resources, and were classified according to the American College of Medical Genetics and Genomics (ACMG)–ClinGen guidelines. [16, 17].

Subsequently, exome sequencing (ES) was performed. Genomic DNA was automatically extracted using the HiPurA Pre‐filled Clinical Multi‐Purpose Nucleic Acid Purification Kit together with the HIMEDIA InstaN Mag‐96 system. DNA concentration was quantified using the Qubit fluorometric system (Thermo Fisher Scientific, United States). ES libraries were prepared using the Roche KAPA HyperExome 96 reaction kit, and sequencing was carried out on the MGI DNBSEQ‐G400 platform. Generated FASTQ files were processed and analyzed using the Genomize SEQ platform (Version 8.7.0; https://seq.genomize.com). Sequence reads were aligned to the human reference genome (GRCh38/hg38), and variant calling, annotation, and filtering were performed through the same platform. Variant interpretation and classification were carried out according to the ACMG guidelines.

### 2.3. Variant Analysis and Classification

Raw sequencing data were analyzed using the Genomize data analysis platform (https://seq.genomize.com). Variant filtering was performed in two main steps to identify potentially pathogenic variants associated with the patient′s clinical phenotype. First, synonymous and noncoding variants were excluded, whereas nonsense, missense, frameshift, splice‐site variants, insertions/deletions (indels), and in‐frame variants were retained for further evaluation. In the second step, variants with a minor allele frequency (MAF) lower than 1.0% in publicly available population databases, including the 1000 Genomes Project (1000G) (https://www.internationalgenome.org/), ExAC (https://exac.broadinstitute.org/), and the Genome Aggregation Database (gnomAD) (https://gnomad.broadinstitute.org/), were selected. Considering the presence of multiple clinical features in the patient, ES data were also systematically evaluated using phenotype‐driven filtering strategies.

Read alignment and variant visualization were performed using the Integrative Genomics Viewer (IGV) (https://software.broadinstitute.org/software/igv/). Identified variants were further investigated using multiple databases, including the Human Gene Mutation Database (HGMD) (https://www.hgmd.cf.ac.uk/), ClinVar (https://www.ncbi.nlm.nih.gov/clinvar/), and the Mastermind Genomic Search Engine (https://mastermind.genomenon.com/), as well as through a comprehensive review of the scientific literature. The potential pathogenicity of candidate variants was assessed using in silico prediction tools, including MutationTaster (https://www.mutationtaster.org/) and Combined Annotation Dependent Depletion (CADD) [18].

**Figure 1:**
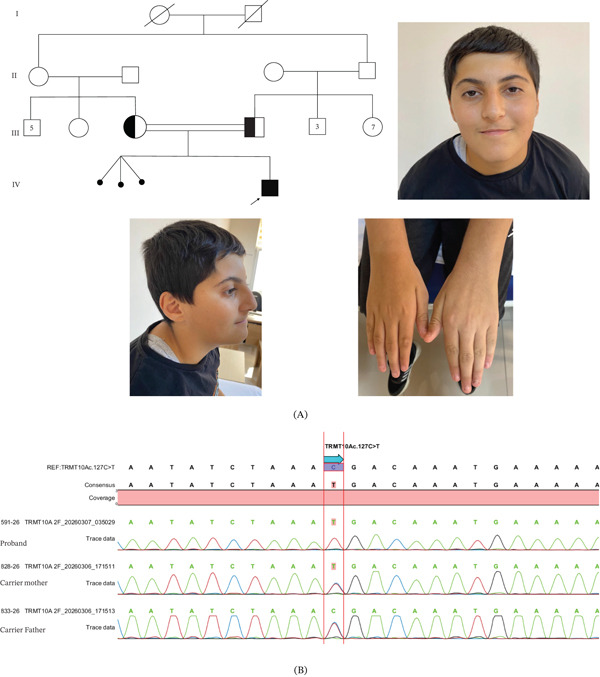
(A) Pedigree of the family and representative clinical photographs of the patient. (B) Sanger sequencing chromatograms confirming the homozygous TRMT10A c.127C > T (p.Arg43Ter) variant in the proband and heterozygous carrier status in both parents.

Variant classification and interpretation were performed according to the ACMG and ClinGen guidelines [16, 17]. Variant confirmation and segregation analyses were subsequently carried out by Sanger sequencing using DNA samples obtained from available family members (Figure 1)B.

Prior to the study, written informed consent was obtained from the legal guardians of the patient for the collection and use of clinical, radiological, and genetic data.

## 3. Results

### 3.1. Case Presentations

The proband was a 15‐year‐old boy who was referred to our clinic because of learning difficulties, poor school performance, and episodes of staring. He was the first living child of healthy Syrian parents who were first‐degree cousins (Figure 1)A. Maternal history revealed a triplet pregnancy that ended at 24 weeks of gestation. The proband was born at 40 weeks of gestation via spontaneous vaginal delivery; however, birth parameters including birth weight, length, and head circumference were not available. A history of neonatal jaundice lasting approximately 1 week was reported.

Neurodevelopmental milestones were delayed compared with peers. He achieved independent sitting at 9 months and independent walking at 24 months. His first single words were spoken after 20 months of age. There was no family history of diabetes mellitus or neurological disease.

On physical examination, his height was 157 cm (−2.3 standard deviations [SD]), weight was 49.5 kg (−1.7 SD), body mass index (BMI) was 20.1 kg/m^2^, and head circumference was 51.5 cm (−3.7 SD). Examination revealed microcephaly, short stature, strabismus, and a buffalo hump. Dysmorphic features included a narrow forehead, thin upper and lower lips, smooth philtrum, broad chin, premaxillary hypoplasia, anteverted and hypoplastic tragus, poorly folded helix, attached earlobes, prominent antihelix root, and tapering fingers (Figure 1)A. His annual growth velocity was 5 cm/year.

Laboratory investigations showed findings consistent with thalassemia minor on complete blood count. Thyroid function tests and routine biochemical parameters were within normal limits. Serum vitamin B12 and vitamin D levels were also normal. Endocrinological evaluation for short stature did not reveal any pathological findings. Fasting plasma glucose was 88 mg/dL (reference range 70–100 mg/dL) and HbA1c was within normal limits. The patient did not report symptoms suggestive of diabetes mellitus such as polyuria, polydipsia, or polyphagia. Bone age assessment using the Greulich–Pyle method was reported as 14 years and 9 months.

Abdominal ultrasonography showed no abnormalities. Brain MRI was also normal. Cardiological evaluation revealed no pathological findings, and both electrocardiography (ECG) and echocardiography (ECHO) were within normal limits. EEG demonstrated generalized high‐amplitude sharp wave discharges lasting 2–3 s. During hyperventilation, two episodes of generalized high‐amplitude sharp wave discharges lasting 3 s and 10 s, respectively, were recorded. Based on these findings, antiepileptic treatment with valproate was initiated. Psychometric evaluation revealed an IQ score of 63, and the proband was diagnosed with mild intellectual disability.

### 3.2. Genetic Findings

Karyotype analysis revealed a normal male karyotype (46,XY); chromosomal microarray analysis did not detect any clinically significant CNVs, and FMR1 CGG repeat expansion testing was negative, excluding Fragile X syndrome.

Subsequently, whole‐exome sequencing (WES) was performed in the proband. The analysis identified a homozygous stop‐gained variant in the *TRMT10A* gene (NM_001375882.1:c.127C > T; NP_001362811.1:p.Arg43Ter) located on chromosome 4q23. The variant was confirmed by Sanger sequencing, which demonstrated that both parents were heterozygous carriers, consistent with an autosomal recessive mode of inheritance (Figure 1A,B).

The identified variant has been reported in the ClinVar database (ClinVar Variation ID: 3343876); however, to the best of our knowledge, no detailed clinical report describing patients harboring this variant has been published in the literature to date. According to the AACMG guidelines, the variant was classified as pathogenic (PVS1, PM2, and PM3). In silico prediction tools also supported a deleterious effect on protein function.

Taken together, our findings describe the clinical and molecular features observed in a patient with a homozygous *TRMT10A* variant.

## 4. Discussion

**Figure 2:**
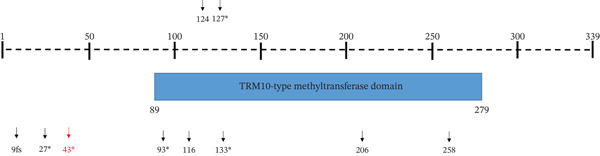
Distribution of reported TRMT10A variants along the protein sequence. The TRMT10A protein (339 amino acids) is shown as a horizontal bar. The catalytic TRM10‐type methyltransferase domain (aa 89–279) is indicated by the shaded box. Variants are plotted according to their positions along the protein sequence. Truncating variants (nonsense and frameshift) are indicated by an asterisk ( ^∗^), whereas missense variants are shown by their amino acid positions. The homozygous stop‐gained variant p.Arg43Ter identified in this study is highlighted in red.

The *TRMT10A* gene has been associated with an autosomal recessive disorder characterized by microcephaly, short stature, and impaired glucose metabolism (OMIM #616013). The distribution of reported *TRMT10A* variants along the protein sequence is illustrated in Figure 2. Notably, a substantial proportion of previously reported variants are located within the catalytic TRM10‐type methyltransferase domain, and missense variants affecting this region are thought to impair the enzymatic activity of *TRMT10A* and consequently disrupt tRNA methylation [8, 9, 19]. In contrast, the homozygous p.Arg43Ter variant identified in our study is located upstream of the catalytic domain and introduces a premature termination codon early in the protein sequence, resulting in marked truncation of the protein. In this context, the variant identified in our study adds to the previously described variant spectrum of *TRMT10A*.

In previously reported cases, early truncating *TRMT10A* variants located upstream of the catalytic domain have also been associated with heterogeneous clinical presentations, particularly regarding metabolic involvement. In the study by Yew et al., a female patient harboring a truncating variant demonstrated diabetes diagnosed at 24 years of age, whereas her affected sibling developed diabetes at 28 years; notably, neither exhibited spontaneous hypoglycemia. Consistent with our case, brain MRI findings were normal, and features such as buffalo hump were also described [14]. In addition, a frameshift variant occurring upstream of the catalytic domain reported by Tokuç et al. was associated with diabetes diagnosed at 15 years of age, normal cranial MRI findings, and drug‐resistant focal epilepsy [20].

Current literature indicates that the age at onset of diabetes in *TRMT10A*‐related syndrome typically ranges between 9 and 22 years, with considerable variability [9]. In some individuals, endocrine manifestations have rarely not been reported. Consistent with our case, Narayanan described two patients aged 12 and 10 years who did not exhibit endocrine abnormalities [11]. In contrast, in the studies by Gillis et al. and Cerealo et al., patients aged 13 and 19 years had not yet developed diabetes and presented only with hypoglycemic symptoms [2, 8]. These observations suggest that endocrine manifestations may emerge later in the disease course and underscore the importance of longitudinal monitoring, even in the absence of early metabolic findings.

The currently documented TRMT10A variant spectrum in the HGMD remains limited, with only 14 variants identified to date, including nine missense/nonsense variants, two splice‐site variants, one small deletion, one small insertion, and one gross deletion. To date, a total of 23 patients harboring *TRMT10A* variants have been described (Table 1) [2, 7–15, 19–23]. The majority of these patients carried homozygous or compound heterozygous loss‐of‐function variants, whereas only one case reported by Zung et al. involved a large deletion encompassing the *TRMT10A* gene at chromosome 4q23 [15]. This observation further supports that a single heterozygous variant is insufficient to explain the phenotype in autosomal recessive disorders and suggests the possible presence of an undetected second variant. Clinically, microcephaly and intellectual disability represent consistent features across all reported cases (23/23, 100%), both of which are also present in our patient (Table 2). Microcephaly has been documented as either congenital with subsequent improvement or persistent, and is frequently accompanied by delayed psychomotor development [2, 7–15, 19–23].

**Table 1:** Reported TRMT10A variants in the literature.

Study	Patient (s)	Nucleotide change	Amino‐acid change	Variant type	Zygosity	Exon/intron	ACMG classification	ClinVar
Igoillo‐Esteve et al. 2013[9]	3	c.379G>A	p.Arg127=	Missense	Hom	Exon 4	VUS	VUS
Gillis et al. 2014[8]	3	c.616G>A	p.Gly206Arg	Missense	Hom	Exon 6	P	P
Zung et al. 2015[15]	1	4q23 deletion	—	CNV	—	—	P	—
Narayanan et al. 2015[11]	2	c.277C>T	p.Arg93Ter	Nonsense	Compound het	Exon 3	P	P/LP
		c.397C>T	p.Arg133Ter	Nonsense		Exon 4	LP	—
Yew et al. 2016[14]	2	c.79G>T	p.Glu27Ter	Nonsense	Hom	Exon 2	P	P
Reuter et al. 2017[21]	2	c.348G>C	p.Lys116Asn	Missense	Hom	Exon 3	VUS	VUS
Boonsawat et al. 2019[19]	1	c.379C>T	p.Arg127Ter	Nonsense	Hom	Exon 4	P	LP/P
Hu et al. 2019[22]	2	c.370C>A	Gln124Lyn	Missense	Hom	Exon 4	VUS	—
Lin et al., 2020[10]	1	c.496–1G>A	—	Splice‐site	Hom	Intron 5	LP	LP
Stern et al. 2021[13]	1	c.616G>A	p.Gly206Arg	Missense	Hom	Exon 6	P	P
Brener et al. 2022[7]	1	c.616G>A	p.Gly206Arg	Missense	Hom	Exon 6	P	P
Şıklar et al. 2023[12]	1	c.379C>T	p.Arg127Ter	Nonsense	Hom	Exon 4	P	P/LP
Samhani et al. 2024[23]	1	c.774A>C	p.Tyr258Ter	Missense	Hom	Exon 8	VUS	—
Ceraolo et al. 2025[2]	1	c.421–1G>A	—	Splice‐site	Hom	Intron 4	LP	—
Tokuç et al. 2024[20]	1	c.23dup	p.Phe9IlefsTer3	Frameshift	Hom	Exon 2	P	P
This study	1	c.127C>T	p.Arg43Ter	Nonsense	Hom	Exon 2	P	P/LP

Abbreviations: CNV, copy number variation; Hom, Homozygous, LP, likely pathogenic; P, pathogenic, VUS, variant of uncertain significance.

**Table 2:** Clinical features of the proband compared with previously reported *TRMT10A* patients.

Clinical feature	Previously reported patients *n* = 23	Proband (this study)
Microcephaly	23/23 (%100)	+
Diabetes	13/17 (%76)	—
Hypoglycemia	2/17 (%12)	—
Delayed pubertal development	7/23 (%30)	—
Epilepsy	12/18 (%67)	+
Intellectual disability	23/23 (%100)	+
Psychomotor development	12/14 (%86)	+
Short stature	17/21 (%81)	+
Buffalo hump	3/18 (%17)	+

Neurologically, epilepsy has been reported in a considerable proportion of patients (12/18, 67%), and the presence of EEG abnormalities and the requirement for antiepileptic treatment in our patient are consistent with these findings [2, 8, 9, 12, 14, 19, 20, 23]. Neuroimaging findings are heterogeneous, with abnormalities observed in approximately half of the documented cases (8/17, 47%), whereas the remaining patients, including ours, exhibit normal brain MRI. Reported abnormalities include periventricular white matter changes [11], pituitary hypoplasia and arachnoid cysts [12], posterior fossa anomalies consistent with Dandy–Walker variant [2], reduced brain volume [7, 9], and brain calcifications [21], indicating the absence of a specific neuroimaging pattern.

Short stature is a common clinical feature (17/21, 81%), although detailed data regarding growth hormone axis evaluation and height SDS remain limited [7–13, 15, 19, 21, 23]. Given the widespread expression of *TRMT10A* in the central nervous system, including the hypothalamus and pituitary gland, its role in growth and endocrine regulation is biologically plausible [12] In addition, the presence of a buffalo hump in our patient, a feature rarely reported in the literature (3/18, 17%) [9, 14, 23], represents a nonspecific clinical finding.

A functional limitation of this report is the absence of experimental validation to confirm the molecular and cellular consequences of the identified *TRMT10A* variant. These findings highlight the importance of cautious interpretation in single‐case studies and emphasize the need for longitudinal clinical evaluation.

## 5. Conclusion

This study describes a patient harboring a homozygous early truncating *TRMT10A* variant (p.Arg43Ter), presenting with microcephaly, intellectual disability, epilepsy, and short stature in the absence of overt metabolic abnormalities at evaluation. Positioned upstream of the catalytic TRM10‐type methyltransferase domain, this variant is predicted to cause loss of function via nonsense‐mediated mRNA decay or production of a truncated, nonfunctional protein, supporting that *TRMT10A*‐related disease is primarily driven by biallelic loss‐of‐function variants rather than being confined to the catalytic domain. This case expands the phenotypic spectrum and indicates that the absence of metabolic features, particularly early in life, does not exclude the diagnosis. The coexistence of core neurodevelopmental features with variably delayed metabolic involvement suggests a dynamic and evolving disease course; therefore, *TRMT10A* should be considered in the differential diagnosis, and careful longitudinal follow‐up, including monitoring of fasting glucose, HbA1c levels, growth velocity, and pubertal development, is warranted to enable early detection of potential metabolic complications.

## Funding

No funding was received for this manuscript.

## Ethics Statement

Ethical approval was obtained from the Ethics Committee of Bandırma Onyedi Eylül University (Decision No.: 26‐20, March 2026).

## Conflicts of Interest

The authors declare no conflicts of interest.

## Data Availability

The data that support the findings of this study are available from the corresponding author upon reasonable request.
